# Genome sequence and virulence factors of a group G *Streptococcus dysgalactiae* subsp. *equisimilis* strain with a new element carrying *erm(B)*

**DOI:** 10.1038/srep20389

**Published:** 2016-02-04

**Authors:** Xiaohui Wang, Xiaoxia Zhang, Zhiyong Zong

**Affiliations:** 1 Center of Infectious Diseases, West China Hospital, Sichuan University, Chengdu, China; 2 Division of Infectious Diseases, State Key Laboratory of Biotherapy, Chengdu, China; 3 Department of Infection Control, West China Hospital, Sichuan University, Chengdu, China

## Abstract

A *Streptococcus dysgalactiae* subsp. *equisimilis* (SDSE) strain WCHSDSE-1, which caused an outbreak of tonsillopharyngitis among healthcare workers in China, was subjected to genome sequencing and analysis. WCHSDSE-1 belongs to the Lancefield group G, *emm* type *stG211.1* and sequence type 44. WCHSDSE-1 has virulence factors for adherence, impairing the recruitment of neutrophils to infection sites and toxins including streptolysins O and S and exotoxin G. WCHSDSE-1 has a 45.4-kb element resembling a conjugative transposon. This element is absent from other known SDSE genomes and contains the macrolide-resistant gene *erm*(B). Conjugative transfer of *erm*(B) was not successful in mating experiments, suggesting that the element might have lost its ability of conjugation. An almost identical element, which contains the tetracycline-resistant gene *tet*(M) instead of *erm*(B), is present on the genome of *Filifactor alocis* ATCC 35896. The boundaries and insertion sites of the two elements were identified and both were flanked by a 3-bp direct repeat, which is characteristic of transposition. In conclusion, the spectrum of virulence factors of WCHSDSE-1 is similar to other SDSE strains causing invasive diseases. WCHSDSE-1 possesses a new transposable element encoding macrolide resistance, which could pick up different resistance genes and could be transferred across species in oral microflora.

*Streptococcus dysgalactiae* subsp. *equisimilis* (SDSE) belongs to *β*-hemolytic streptococci. Although it has long been considered as a nonpathogenic colonizer of human throat and skin, SDSE is now recognized a causative agent of both human and livestock infections^1^. Like *Streptococcus pyogenes*, a well-known pathogen of group A streptococci, SDSE can cause a wide spectrum of human diseases from localized infection such as pharyngitis and pyoderma to invasive life-threatening infections such as bacteremia and toxin-mediated streptococcal toxic shock syndromes^1^. According to a widely-adopted classification scheme^2^, SDSE is classified into Lancefield group G (about 3/4), C (about 1/4) or A or L (occasionally). Besides SDSE, group G streptococci also include *Streptococcus anginosus*, *Streptococcus canis* and *Streptococcus intestinalis*^1^. Several outbreaks of pharyngitis or tonsillopharyngitis have been attributed to group G streptococci^3^,^4^,^5^,^6^,^7^, although the isolates have not been identified to the species level in these reports.

In August 2013, an outbreak of tonsillopharyngitis caused by SDSE among 30 healthcare workers (HCWs) occurred in a municipal hospital in China. Group G streptococci were recovered from the throat swabs of all of the 30 HCWs. All streptococci isolates were further identified as SDSE and were found to belong to the same strain (see below). One of the 30 isolates was randomly selected for whole genome sequencing and is reported here.

## Results and Discussion

### All SDSE isolates belonged to the same ST44-*stG211.1* strain

All 30 isolates were identified as SDSE and belonged to the same *emm* type (*stG211.1*), the same sequence type (ST44), and the same pulsotype (unpublished data). The above findings suggest that the isolates represented a single strain. Among the 30 isolates, one, designated WCHSDSE-1, was selected for whole genome sequencing.

### Genome sequence of SDSE isolate WCHSDSE-1

A total of 5,068,234 reads were obtained from the genome sequencing of WCHSDSE-1 comprising 506,823,400 bases with a 39.5% GC content. Reads were assembled into 70 contigs that were ≥500 bp in length (N50 = 63,425 bp) and contained 2,083,980 bp nucleotides. WCHSDSE-1 contained 2,076 protein coding sequences (CDS), 60 tRNA and 1 tmRNA.

### ST44 corresponds to multiple *emm* types

*In silico* multi-locus sequence typing (MLST) confirmed that WCHSDSE-1 belonged to ST44, a sequence type which has not been found in many countries but has been reported in India and Australia before^8^,^9^. A single ST may correspond to multiple *emm* types, which represent serospecificities of *S. pyogenes* and possibly of group G and C streptococci such as SDSE. In a previous study, ST44 of SDSE was associated with five *emm* types (*stC36*, *stG245*, *stG480*, *stG6* and *stGL265*)^9^, none of which is the *stG211.1* type seen here. A SDSE isolate of *stG211.1* has been previously recovered from a patient with pharyngitis in Vellore, India in 2007 but the ST of the isolate was not provided (the CDC *emm* database, ftp://ftp.cdc.gov/pub/infectious_diseases/biotech/tsemm/stG211.1.sds). In addition, a previous investigation on group G SDSE showed significant clonal diversity among SDSE in Chinese children^10^ but none of the isolates were *stG211.1*. Therefore, we believe that this is the first report of a ST44-*stG211.1 *SDSE isolate, which appears to be uncommon.

### WCHSDSE-1 is close to a SDSE from Sweden by phylogenetic analysis.

**Figure 1:**
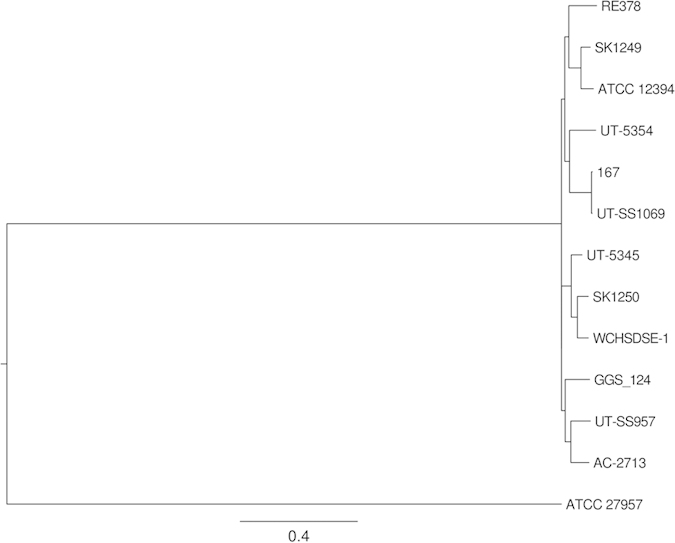
Phylogenetic tree of SDSE based on whole genome sequences. Strains (GenBank accession number, genome statue [complete or draft]) are GGS_124 (AP010935, complete), ATCC 12394 (NC_017567, complete), RE378 (NC_018712, complete), AC-2713 (NC_019042, complete), 167 (NC_022532, complete), SK1250 (NZ_AFUL01000000, draft), ATCC 27957 (NZ_CM001076, only chromosome complete), SK1249 (NZ_AFIN01000000, draft), UT-5345 (NZ_LAKV01000000, draft), UT-5354 (NZ_LAKU01000000, draft), UT-SS957 (NZ_LAKT01000000, draft), UT-SS1069 (NZ_LAKS01000000, draft). ATCC 27957 is a SDSD strain and is included as an outgroup.

Based on the phylogenetic analysis, WCHSDSE-1 is most closely related to strain SK1250 (CCUG 45841; Fig.1), which belongs to Lancefield group A and *emm* type *stG840* and was isolated from human throat in Sweden in 2001 ( http://www.straininfo.net/strains/330680/browser). The relative closeness between WCHSDSE-1 and SK1250 was also confirmed by *in silico* genome-to-genome comparisons (Table 1). Both phylogenetic and genome-to-genome analyses confirmed that the SDSE strains clustered together more closely in comparison to the *S. dysgalactiae* subsp. *dysgalactiae* (SDSD) strain ATCC 25957.

### The virulence factor profile of WCHSDSE-1 is largely similar to other SDSE causing invasive diseases

The virulence factors of SDSE have been well characterized but were determined in strains causing invasive diseases rather than those causing tonsillopharyngitis. WCHSDSE-1 possessed a few virulence factors including: *lmb*, *pavA*, *gfbA* and *gapC* for adherence; *scpA* and *scpB* encoding complement proteases involved in an impaired recruitment of neutrophils to infection sites; determinants of streptolysins O and S and exotoxin G and genes encoding streptokinases (Table 2). Of note, superantigen G and streptolysin S genes that are regarded as the most important virulence factors causing invasive diseases^11^ are also present in WCHSDSE-1. On the other hand, the *fnB* gene, which encodes a fibronectin binding protein, is present in most SDSE strains causing invasive diseases but is absent from WCHSDSE-1. In contrast, WCHSDSE-1 possesses another fibronectin binding protein-encoding gene, *gfbA*, that is absent from most SDSE strains causing invasive diseases. The exact roles of *fnB* and *gfbA* of SDSE in causing invasive or non-invasive diseases remain undetermined but warrant further investigation. Most (79/88) of the proposed virulence factors, which have not been experimentally characterized, of the invasive strain GGS_124 are also present on WCHSDSE-1 (Table S1 in the Supplementary file). Among the 9 factors absent from WCHSDSE-1, two, i.e. *sdn* (encoding a deoxyribonuclease) and an unnamed gene (locus_tag 0157 of GGS_124, encoding a fimbrial subunit protein), are also absent from all other SDSE strains and therefore represent strain-specific factors for strain GGS_124. The remaining seven factors are either absent from at least one strain causing invasive diseases or present in some non-invasive strains. The spectrum of virulence factors of WCHSDSE-1 is therefore largely similar to SDSE strains causing invasive diseases (Table 2). Based on analysis here, invasive and non-invasive strains can not be distinguished by the spectrum of virulence factors.

As virulence factors may be carried by prophages, sequences belong to prophages were predicted from the genome sequence of WCHSDSE-1. Two prophages were identified, one of which is 37.6 kb and shows 94% identity (67% coverage) with the phage 3396 in a SDSE isolate (GenBank accession no. EF207558)^12^. Another prophage is 38.2 kb and shows 96% identity (45% coverage) with the phage T12 in *Streptococcus pyogenes* (GenBank accession no. KM289195). Of note, none of the virulence factors identified are present on either of the two prophages.

### The macrolide-resistant gene *erm*(B) was carried by a new transposable element in WCHSDSE-1

Isolate WCHSDSE-1 haboured only one known antimicrobial resistance gene, which is *erm*(B). *erm*(B) is able to mediate resistance to macrolides, clindamycin and streptogramins B^13^. WCHSDSE-1 was susceptible to penicillin, cefotaxime, levofloxacin and vancomycin but was resistant to erythromycin, azithromycin, clindamycin and tetracycline. The presence of *erm*(B) corresponds to the macrolide and clindamycin resistance phenotype of WCHSDSE-1.

**Figure 2:**
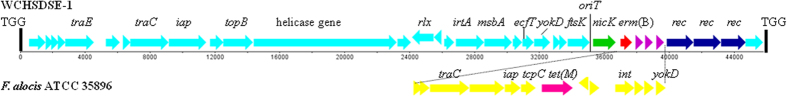
The conjugative transposon-like elements in WCHSDSE-1 and *F. alocis* ATCC 35896. Different regions of the two elements are indicated by dotted lines. Boundaries of the transposon are indicated by black poles and the 3-bp DR (TGG) are shown. Genes and their product or function are listed: *traE*, conjugal coupling protein; *traC*, type-IV secretion system protein; *iap*, endopeptidase p60 precursor; *topB*, DNA topoisomerase 3; helicase gene, helicase/DNA methylase; *rlx*, relaxase/mobilisation nuclease domain protein; *irtA*, iron import ATP-binding/permease protein; *msbA*, putative ABC transporter ATP-binding protein; *ecfT*, energy-coupling factor transporter transmembrane protein; *ykoD*, putative HMP/thiamine import ATP-binding protein; *ftsK*, DNA translocase; *nicK*, relaxase; *erm*, macrolide resistance; *rec*, recombinase; *tcpC*, conjugative transposon protein; *tet(M)*, tetracycline resistance; *int*, integrase; other genes are of unknown function. The scale in bp is shown.

When the genetic context of *erm*(B) was examined, we found that a large 45.4-kb region containing *erm*(B) in WCHSDSE-1 was absent from other SDSE genomes. However, an almost identical region is present on the genome of *Filifactor alocis* ATCC 35896 (GenBank accession number CP002390). *F. alocis* is an oral Gram-positive anaerobic rod that can cause periodontal diseases^14^. However, the region of *F. alocis* ATCC 35896 has a 14.1-kb sequence containing the tetracycline resistance gene *tet*(M) in place of the 1.5-kb sequence containing *erm*(B) seen in isolate WCHSDSE-1 (Fig. 2). This 45.4-kb region of isolate WCHSDSE-1 appears to be a conjugative transposon as it contains genes encoding the site-specific recombinase, a relaxase-encoding gene (*nicK*), an origin of transfer (*oriT*; ACCCCCCGTATCTAACAGGGGGGT [inverted repeats are underlined], identical to that of the well-characterized conjugative transposon Tn*916*) upstream of *nicK*, and a gene encoding conjugal transfer coupling protein. Of note, there are three recombinase-encoding genes, which cluster together (Fig. 2), and therefore it is reasonable to hypothesize that they may encode a single recombinase through frameshift. Mating experiments were performed to examine whether the putative transposon was conjugative. However, despite repeated attempts, conjugative transfer of *erm*(B) to the recipient strain was not detected. This suggests that the putative transposon carrying *erm*(B) in strain WCHSDSE-1 may have lost its ability of conjugation or the conjugation occurred in a rate lower than that could be detected in the mating experiments.

This putative transposon was present in a single copy in both isolate WCHSDSE-1 and *F. alocis* ATCC 35896. Comparisons to other SDSE genome sequences allowed us to determine the exact boundary and the insertion site of this putative transposon. A 3-bp direct repeat (DR, Fig. 2) was identified flanking the transposon in isolate WCHSDSE-1 and the corresponding element in *F. alocis* ATCC 35896. In WCHSDSE-1, this putative transposon was inserted into a spacer region between two hypothetical open reading frames of unknown function (locus tags GGS_0498 and GGS_0499 in strain RE378; SDSE167_0576 and SDSE167_0577 in strain 167; SDEG_0522 and SDEG_0523 in strain GGS_124). The boundary of the putative transposon identified from isolate WCHSDSE-1 was also verified in *F. alocis* ATCC 35896. The putative transposon is also flanked by a 3-bp DR (Fig. 2) in *F. alocis* ATCC 35896. The evidence of transposition characterized by the presence of 3-bp DR suggests that the putative transposons in strain WCHSDSE-1 and *F. alocis* ATCC 3589 were truly transposable elements. The presence of highly similar transposable elements present in two species of human oral microflora suggests that interspecies transfer of a common transposon might have occurred among oral microflora.

## Conclusions

We report the genome sequence of a *stG211.1*-ST44 SDSE strain WCHSDSE-1 causing tonsillopharyngitis. Isolate WCHSDSE-1 had a nearly identical spectrum of virulence factors with SDSE strains causing invasive diseases, suggesting that strains causing invasive and non-invasive diseases may not be distinguished by the presence or absence of certain virulence factors. A new putative transposon encoding macrolide resistance was identified. This putative transposon is likely to be acquired from species other than SDSE in the oral microflora and is able to pick up different antimicrobial resistance genes.

## Methods

Species identification was performed by partially sequencing the 16S rRNA gene as described previously^15^. Three methods were used for strain typing: the *emm* (encoding M protein) genotyping scheme ( http://www.cdc.gov/streplab/protocol-emm-type.html), MLST ( http://sdse.mlst.net/) and pulsed field gel electrophoresis (PFGE)^16^.

Genomic DNA of isolate WCHSDSE-1 was prepared using the QIAamp DNA Mini Kit (Qiagen, Hilden, Germany) and was subjected to whole genome sequencing with a ca. 100 × coverage using the Hiseq 2500 Sequencer (Illumina, San Diego, CA, USA) following the manufacturer’s protocol at the Beijing Genomics Institute. Reads were assembled into contigs using the Spades program^17^ and the Prokka program^18^ was employed for annotating the genomic sequence. *In silico* MLST was performed using the MLST tool of Centre for Genomic Epidemiology. Prophages were predicted using the Prophinder program^19^, while known antimicrobial resistance genes were predicted using the ResFinder tool of Centre for Genomic Epidemiology.

A total of 11 SDSE genomes (5 complete and 6 draft versions) were available in GenBank ( http://www.ncbi.nlm.nih.gov/genome/genomes/823; accessed on June 17, 2015). The phylogenetic relatedness of WCHSDSE-1 and the 11 SDSE strains was investigated based on genome sequences by the Harvest suite^20^ and SDSD type strain ATCC 27957^20^ was included as an outgroup. *In silico* inter-genomic comparisons between WCHSDSE-1 and the 11 SDSE strains were performed using GGDC (formula 2) which mimics DNA-DNA hybridization (DDH)^21^. Potential unique genes were identified using the Gegenees program^22^ with a stringent threshold of a 0.9 or higher score generated by Gegenees.

A dataset of virulence factors of SDSE was established by retrieving the corresponding sequences (Table 2) from the Virulence Factors of Pathogenic Bacteria database (VFDB, http://www.mgc.ac.cn/cgi-bin/VFs/) and the review of Brandt *et al.*^1^ An additional dataset was compiled using the 88 known or putative virulence factors of SDSE strain GGS_124, which was demonstrated to cause streptococcal toxic shock syndrome^23^. The assembled contigs of WCHSDSE-1 were aligned against the datasets using the BLAST program. The presence of a virulence factor in WCHSDSE-1 was defined using the cutoff value ≥60% coverage and ≥70% nucleotide identity.

*In vitro* susceptibility of isolate WCHSDSE-1 to β-lactams (penicillin and cefotaxime), clindamycin, macrolides (erythromycin and azithromycin), levofloxacin, tetracycline and vancomycin were determined by disk diffusion following the recommendations of CLSI^24^.

Both filter-based^25^,^26^ and broth-based mating methods^27^ were used for conjugation experiments as described previously. A levofloxacin-resistant but erythromycin-susceptible *Streptococcus agalactiae* clinical isolate was used as the recipient. Transconjugants were selected on agar plates containing 1 μg/ml erythromycin and 8 μg/ml levofloxacin.

## Additional Information

**Accession Codes**: Sequence reads and the Whole Genome Shotgun project of WCHSDSE-1 was deposited into DDBJ/EMBLGenBank under accession SRR2049024 and LDYC00000000,respectively. The new putative conjugation transposon was deposited into DDBJ/EMBL/GenBank under accession KT005459.

**How to cite this article**: Wang, X. *et al.* Genome sequence and virulence factors of a group G *Streptococcus* *dysgalactiae* subsp. *equisimilis* strain with a new element carrying *erm(B)*. *Sci. Rep.* **6**, 20389; doi: 10.1038/srep20389 (2016).

## Supplementary Material

Supplementary Information

## Figures and Tables

**Table 1:** *In silico* genome-to-genome comparison between WCHSDSE-1 and other SDSE strains by GGDC^a^.

Query genome	Reference genome	DDH (%)	Distance
WCHSDSE-1	SK1250	94.6	0.0071
	UT-5345	92.6	0.0093
	GGS_124	91.5	0.0105
	UT-SS1069	91	0.011
	SK1249	90.9	0.0112
	167	90.7	0.0113
	UT-5354	90.7	0.0114
	RE378	90.1	0.0121
	AC-2713	90	0.0122
	UT-SS957	90	0.0122
	ATCC 12394	89.3	0.0129
	ATCC 27957^a^	67.8	0.0392

^a^ATCC 27957 is a SDSD strain and was included as an outgroup.

**Table 2:** Virulence factors of SDSE strains with whole genome sequences available.

Function	Gene	Product	Accession no.	SDSE strains^a^												SDSD^b^
				WCHSDSE-1	GGS_124	ATCC 12394	RE378	AC-2713	167	UT-5345	UT-5354	SK1250	SK1249	UT-SS957	UT-SS1069	ATCC 27957^c^
Adherence	*fbsA*	fibrinogen-binding protein	AJ421083	−	−	−	−	−	−	−	−	−	−	−	−	−
	*lmb*	laminin-binding surface protein	NC_004116	+	+	+	+	+	+	−	−	+	+	−	−	−
	*pavA*	adherence and virulence protein A	NC_004116	+	+	+	+	+	+	−	−	+	+	−	−	+
	*fnbA*	fibronectin binding protein	Z22150	−	−	−	−	−	−	−	−	−	−	−	−	+
	*fnbB*	fibronectin binding protein	Z22151	−	−	−	−	−	−	−	−	−	−	+	−	+
	*fnB*	fibronectin binding protein	Z29088	−	+	+	+	+	+	−	−	+	−	+	+	−
	*gfbA*	fibronectin binding protein	AJ605744	+	−	−	+	−	−	−	−	−	−	−	−	−
	*gapC*	glyceraldehyde 3−P dehydrogenase	AF375662	+	+	+	+	+	+	−	−	+	+	+	+	+
Antiphagocytosis	*cba*	C protein β antigen	AB121739	−	−	−	−	−	−	−	−	−	−	−	−	−
	*cpsA-B-C-D-E-F-G-H-J-K-L-M-N-O*	capsular	NC_004116	−	−	−	−	−	−	−	−	−	−	−	−	−
	*neuA-B-C-D*	capsular	NC_004116	−	-	−	−	−	−	−	−	−	−	−	−	−
Complement protease	*scpA*	C5a peptidase	AP010935	+	+	+	+	+	+	+	−	+	+	−	−	+
	*scpB*	C5a peptidase	WP_001227855	+	+	+	+	+	+	+	−	+	+	−	−	+
Exoenzyme	*hylB*	hyaluronate lyase	NC_004116	−	−	−	−	−	−	−	−	−	−	−	−	−
Invasion	*bca*	C protein α antigen	M97256	−	−−	−	−	−	−	−	−	−	−	−	−	−
Toxin	*cylE*	β-haemolysin/cytolysin	NC_004116	−	−	−	−	−	−	−	−	−	−	−	−	−
	*cfb*	CAMP factor	NC_004116	−	−	−	−	−	−	−	−	−	−	−	−	−
	*slo*	streptolysin O	AB128035	+	+	+	+	+	+	−	−	+	+	−	−	−
	*sagA-B-C-D-E-F-G-H-I*	streptolysin S	AP010935	+	+	+	+	+	+	−	−	+	+	−	−	+
	*speA*	exotoxin A	AY049745	−	−	−	−	−	−	−	−	−	−	−	−	−
	*speC*	exotoxin C	GQ923929	−	−	−	−	−	−	−	−	−	−	−	−	−
	*speM*	exotoxin M	AJ557010	−	−	−	−	−	−	−	−	−	−	−	−	−
	*speG*	exotoxin G	AJ489606	+	+	−	−	+	+	−	−	+	−	+	−	−
	*smeZ*	exotoxin Z	GQ923928	−	−	−	−	−	−	−	−	−	−	−	−	−
Spreading	*skc*	streptokinase	AP012976	+	+	+	+	+	+	−	−	+	+	−	−	−
	*skg*	streptokinase	CP002215	+	+	+	+	+	+	−	−	+	+	−	−	−

^a^SDSE strains were all human clinical isolates; strain names in bold were those isolated from blood or causing invasive diseases. The source of UT-SS957 (underlined) remains unknown.

^b^SDSD strain ATCC 27957 caused a bovine udder infection.
