# Supplementary material for: Genome sequence and virulence factors of a group G Streptococcus dysgalactiae subsp. equisimilis strain with a new element carrying erm(B)
Source: Sci Rep. 2016 Feb 4;6:20389. doi: 10.1038/srep20389 (PMC4740735; doi:10.1038/srep20389)
Supplement: Supplementary Information [file srep20389-s1.pdf]

Genome sequence and virulence factors of a group G *Streptococcus dysgalactiae* subsp. *equisimilis* strain with a new element carrying *erm(B)*

Xiaohui Wang[1,2], Xiaoxia Zhang[1,2], Zhiyong Zong[1, 2, 3 §]

[1] *Center of Infectious Diseases, West China Hospital, Sichuan University, Chengdu, China.*

[2] *Division of Infectious Diseases, State Key Laboratory of Biotherapy, Chengdu, China.*

[3] *Department of Infection Control, West China Hospital, Sichuan University, Chengdu, China.*

Supplementary file

Table S1. The presence of known or putative virulence factors of strain GGS_124 on other SDSE strains with whole genome sequences available.

Gene	Locus tag	Product name	SDSE strains[a]											SDSD[b]
			WCHSDSE-1	**ATCC 12394**	**RE378**	**AC-2713**	**167**	SK1250	SK1249	**UT-5345**	**UT-5354**	UT-SS957	UT-SS1069	ATCC 27957
hlyX	0427	Putative hemolysin	+	+	+	+	+	+	+	+	-	-	-	+
sagA	0705	Streptolysin S precursor	+	+	+	+	+	+	+	-	-	-	-	-
ebsA	0776	Pore forming protein	+	+	+	-	+	+	+	-	-	-	-	+
hlyIII	1015	HlyIII, hemolysin	+	+	+	+	+	+	+	-	-	-	-	+
hlyA1	1483	Hemolysin	+	+	+	+	+	+	+	-	-	-	-	+
slo	2027	Streptolysin O precursor	+	+	+	+	+	+	+	-	-	-	-	-
speG	1991	Exotoxin G variant 4	+	-	-	+	+	+	-	-	-	+	-	-
ska	0233	Streptokinase	+	+	+	+	+	+	+	-	-	-	-	-
	0342	Membrane protease protein family	+	+	+	+	+	+	+	-	-	-	-	+
prtS	0468	Truncated cell envelope proteinase	+	+	+	+	+	+	+	+	-	-	-	+
cspA	0574	Truncated cell-surface protease	+	+	+	+	+	+	+	-	-	-	-	+
	0604	Putative peptidase	+	+	+	+	+	+	+	-	-	-	-	+
	0605	Peptidase family U32	+	+	+	+	+	+	+	-	-	-	-	+
pepD	0673	Dipeptidase	+	+	+	+	+	+	+	-	-	-	-	+
scpA	0933	Streptococcal C5a peptidase	+	+	+	+	+	+	+	-	-	-	-	-
	1286	Putative exfoliative toxin	+	+	+	+	+	+	+	-	-	-	-	+
	1538	ATP-dependent endopeptidase	+	+	+	+	+	+	+	-	-	-	-	+
cppA	1906	Putative C3-degrading proteinase	+	+	+	+	+	+	+	-	-	+	+	+
degP	2177	Endopeptidase	+	+	+	+	+	+	+	-	-	-	-	+
	0161	Fibronectin binding protein	+	+	+	+	+	+	+	-	-	-	-	-
stg480.0	0230	Antiphagocytic M protein	+	+	+	+	+	+	+	-	-	-	-	-
pulA	0237	Pullulanase LPQTG	+	+	+	+	+	+	+	-	-	-	-	+
eno	0704	Phosphopyruvate hydratase	+	+	+	+	+	+	+	-	-	-	-	+
lmb	0935	Laminin binding protein	+	+	+	+	+	+	+	-	-	-	-	-
fbp	1263	Fibronectin binding protein	+	+	+	+	+	+	+	-	-	-	-	+
spg	1358	Immunoglobulin G binding protein	+	+	+	+	+	+	+	-	-	-	-	+

inlA	1372	Internalin protein	+	+	+	+	+	+	+	-	-	-	-	+
cbp	1781	Putative collagen binding protein	-	-	+	-	-	-	-	-	-	-	-	-
plr	1936	Glyceraldehyde-3-phosphate dehydrogenase, plasmin receptor	+	+	+	+	+	+	+	-	-	+	+	+
	1984	Fibronectin binding protein	+	+	+	+	+	+	+	-	-	+	+	+
hylB	0654	Hyaluronate lyase precursor	+	+	+	+	+	+	+	-	-	-	-	+
hyl	1588	Hyaluronoglucosaminidase	+	+	+	+	+	+	+	-	-	-	-	-
fhuGBDC	0431-0434	Ferrichrome transporter	+	+	+	+	+	+	+	+	-	-	-	+
mtsABC	0488-0490	Metal transporter	+	+	+	+	+	+	+	+	-	-	-	+
fhuDGC	1860-1862	Ferrichrome transporter	+	+	+	+	+	+	+	-	+	-	+	+
sda1	0541	Putative streptodornase	+	+	+	+	+	+	+	-	-	-	-	+
	0714	Extracellular nuclease	+	+	+	+	+	+	+	-	-	-	-	+
endA	0732	DNA-entry nuclease	+	+	+	+	+	+	+	-	-	-	-	+
	0825	Putative cell surface 5'-nucleotidase	+	+	+	+	+	+	+	-	-	-	-	+
sdn	1103	Deoxyribonuclease	-	-	-	-	-	-	-	-	-	-	-	-
	0256	PTS system, IIB component	+	+	+	+	+	+	+	-	-	-	-	+
	0322	Hypothetical membrane spanning protein	+	+	+	+	+	+	+	-	-	-	-	+
tig	0327	Trigger factor	+	+	+	+	+	+	+	-	-	-	-	+
oppA	0356	Oligopeptide binding protein	+	+	+	+	+	+	+	-	-	-	-	+
	0429	Putative manganese-dependent inorganic pyrophosphatase	+	+	+	+	+	+	+	+	-	-	-	+
	0502	Surface antigen	+	+	+	+	+	+	+	+	-	-	-	+
	0503	67 kDa Myosin-crossreactive antigen	+	+	+	+	+	+	+	+	-	-	-	+
	0618	Hypothetical protein	+	+	-	+	+	+	+	-	-	-	-	+
	0619	Glutathione peroxidase	+	+	+	+	+	+	+	-	-	-	-	+
atpF	0725	ATP synthase B chain	+	+	+	+	+	+	+	-	-	-	-	+
	0924	Periplasmic component of efflux system	+	+	+	+	+	+	+	-	-	-	-	-
isp.1	0929	Immunogenic secreted protein	+	+	+	+	+	+	+	-	-	-	-	-
	1113	Collagen-like protein	-	-	-	-	-	-	-	-	+	-	-	-
	1216	Hypothetical membrane spanning protein	+	+	+	+	+	+	+	-	-	-	-	+

lepA	1222	GTP binding protein	+	+	+	+	+	+	+	-	-	-	-	+
	1239	Hypothetical membrane associated protein	+	+	+	+	+	+	+	-	-	-	-	+
aphA	1259	Acid phosphatase, class B	+	+	+	+	+	+	+	-	-	-	-	-
	1285	Hypothetical protein	+	+	+	+	+	-	+	-	-	-	-	-
dltA	1309	D-alanine-poly(phosphoribitol) ligase subunit1	+	+	+	+	+	+	+	-	-	-	-	+
arcC	1542	Carbamate kinase	+	+	+	+	+	+	+	-	-	-	-	+
arcA	1548	Arginine deiminase	+	+	+	+	+	+	+	-	-	-	-	+
msrA.1	1555	Bifunctional methionine sulfoxide reductase A/Bprotein	+	+	+	+	+	+	+	-	-	-	-	+
	1581	Sugar binding protein	+	+	+	+	+	+	+	-	-	-	-	-
	1698	Putative phosphohydrolase	+	+	+	+	+	+	+	-	-	-	-	+
isp.2	1865	Immunogenic secreted protein	+	+	+	+	+	+	+	-	+	-	+	+
lppC	1935	Acid phosphatase	+	+	+	+	+	+	+	-	-	+	+	+
prgA	1940	Surface exclusion protein	+	+	+	+	+	+	+	-	-	+	+	+
hasC.2	1980	UDP-glucose pyrophosphorylase	+	+	+	+	+	+	+	-	-	+	+	+
nga	2029	NADase	+	+	+	+	+	+	+	-	-	-	-	-
	0157	fimbrial subunit protein	-	-	-	-	-	-	-	-	-	-	-	-
	0158	T-antigen-like fimbrial structural subunit protein	+	+	-	-	-	-	+	-	-	-	-	-
	0180	hypothetical protein	-	+	+	+	+	+	+	-	-	-	-	+
	0267	hypothetical membrane associated protein	+	+	+	+	+	+	+	-	-	-	-	+
	0805	cell wall surface anchor family protein	+	+	-	+	+	+	+	-	-	-	-	+
	0918	histidine triad protein	+	+	+	+	+	+	+	-	-	-	-	+
	0932	complement inhibitor protein	-	-	+	-	+	-	-	-	-	-	-	-
	0979	nisin resistance protein, putative	+	+	+	+	+	+	+	-	-	-	-	+
	1327	YaeC family protein	+	+	+	+	+	+	+	-	-	-	-	-
	1429	probable surface antigen negative regulator	+	+	+	+	+	+	+	-	-	-	-	-
	1480	hypothetical protein	+	+	+	+	+	+	+	-	-	-	-	+
	1511	hypothetical protein	+	+	+	+	+	+	+	-	-	-	-	+
	1573	adhesion protein	-	+	+	+	+	+	+	-	-	-	-	-
	1601	hypothetical protein	-	+	+	+	-	-	+	-	-	-	-	-
	1773	hypothetical protein	-	-	+	-	+	+	-	-	+	-	-	-
	1969	hypothetical protein	+	+	+	+	+	+	+	-	-	+	-	+
	1984	protein F2-like fibronectin binding protein	+	+	+	+	+	+	+	-	-	+	+	+

2022	hypothetical protein	+	+	+	+	+	+	+	-	-	-	-	+
2141	hypothetical protein	+	+	-	+	-	-	-	-	-	-	-	-

[a] SDSE strains were all from human. Strain names in bold were those isolated from blood or causing invasive diseases. The source of UT-SS957 (underlined) remains unknown.

[b] SDSD strain ATCC 27957 caused bovine udder infection.
